# VEGF-A splice variants bind VEGFRs with differential affinities

**DOI:** 10.1038/s41598-020-71484-y

**Published:** 2020-09-02

**Authors:** Spencer B. Mamer, Ashley Wittenkeller, P. I. Imoukhuede

**Affiliations:** 1 Department of Bioengineering, University of Illinois at Urbana-Champaign, Urbana, IL USA; 2 Department of Biomedical Engineering, Washington University in St. Louis, St. Louis, MO USA

**Keywords:** Growth factor signalling, Angiogenesis, Systems biology

## Abstract

Vascular endothelial growth factor A (VEGF-A) and its binding to VEGFRs is an important angiogenesis regulator, especially the earliest-known isoform, VEGF-A_165a_. Yet several additional splice variants play prominent roles in regulating angiogenesis in health and in vascular disease, including VEGF-A_121_ and an anti-angiogenic variant, VEGF-A_165b_. Few studies have attempted to distinguish these forms from their angiogenic counterparts, experimentally. Previous studies of VEGF-A:VEGFR binding have measured binding kinetics for VEGFA_165_ and VEGF-A_121_, but binding kinetics of the other two pro- and all anti-angiogenic splice variants are not known. We measured the binding kinetics for VEGF-A_165_, -A_165b_, and -A_121_ with VEGFR1 and VEGF-R2 using surface plasmon resonance. We validated our methods by reproducing the known affinities between VEGF-A_165a_:VEGFR1 and VEGF-A_165a_:VEGFR2, 1.0 pM and 10 pM respectively, and validated the known affinity VEGF-A_121_:VEGFR2 as K_D_ = 0.66 nM. We found that VEGF-A_121_ also binds VEGFR1 with an affinity K_D_ = 3.7 nM. We further demonstrated that the anti-angiogenic variant, VEGF-A_165b_ selectively prefers VEGFR2 binding at an affinity = 0.67 pM while binding VEGFR1 with a weaker affinity—K_D_ = 1.4 nM. These results suggest that the − A_165b_ anti-angiogenic variant would preferentially bind VEGFR2. These discoveries offer a new paradigm for understanding VEGF-A, while further stressing the need to take care in differentiating the splice variants in all future VEGF-A studies.

## Introduction

The vascular endothelial growth factors have been extensively studied as signaling molecules in angiogenesis^1,2^, and their signaling comprises several components. The mammalian VEGF family includes five homodimeric ligands: VEGF-A, VEGF-B, VEGF-C, VEGF-D, and placental growth factor (PlGF)^3^. VEGF-A signal transduction unfolds following the pattern common to other tyrosine kinase receptors (RTKs), like the fibroblast growth factor (FGF) and platelet-derived growth factor (PDGF) families: (1) ligands bind a receptor monomer, promoting dimerization with another free receptor; (2) phosphorylation occurs at specific tyrosine residues depending on conformational changes allowed by the ligand—i.e. signaling is not directly coupled to binding, but dependent on ligand structure^4,5^; (3) adaptor proteins bind these tyrosine residues and undergo phosphorylation^6^, and (4) phosphorylated adaptor proteins initiate effector signaling cascades^7^ that can ultimately mediate cell-level responses such as cell migration, proliferation, and cell survival^8^.

**Figure 1:**
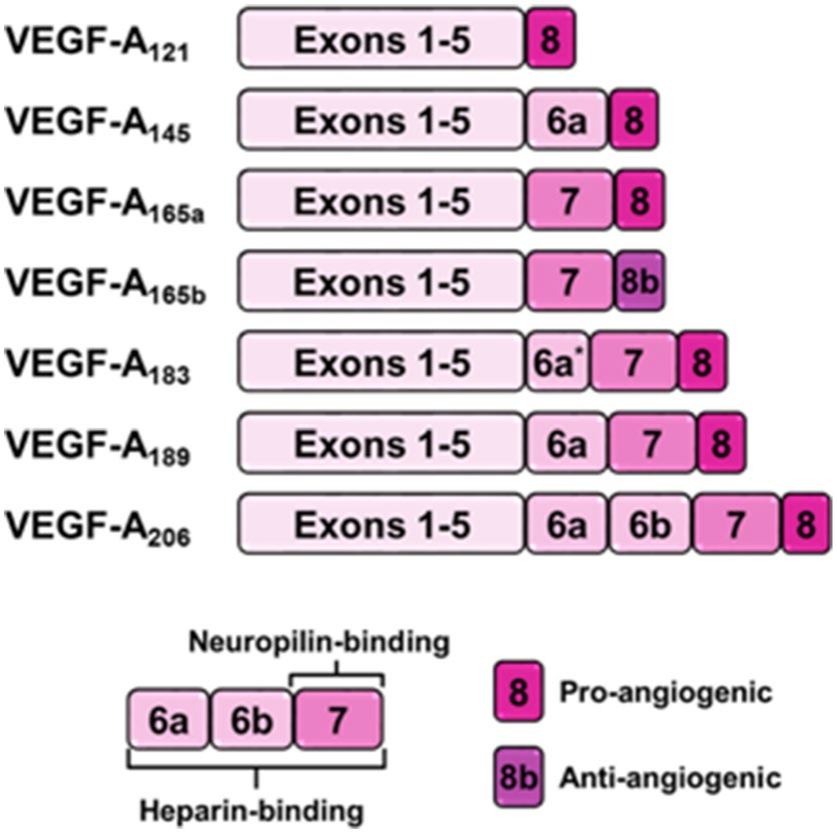
Structural and functional differences of VEGF-A alternative splice variants.

The most well-studied ligand is VEGF-A^9^. VEGF-A has a wide array of isoforms produced through alternative mRNA splicing^10,11^, including: VEGF-A_121_, VEGF-A_121_b, VEGF-A_145_, VEGF-A_145_b, VEGF-A_165_, VEGF-A_165_b, VEGF-A_183_, VEGF-A_189_, and VEGF-A_206_ (Fig. 1), in addition to VEGF-A_111_, an abnormal splice variant induced by genotoxic stressors^12^. Members of the VEGF-A_xxx_ family are pro-angiogenic, whereas those designated as VEGF-A_xxx_b have been described as ‘anti-angiogenic’ due to their less-effective activation of VEGFRs^1,13^, but are more accurately described as weak VEGFR2 agonists^14^. Additionally, VEGF-A_xxx_b splice variants cannot bind heparin-sulfate chains or the VEGFR2 coreceptor, neuropilin-1^15^. Given this modulatory signaling role, several of these VEGF-A_xxx_b isoforms have been proposed as targets for treating such angiogenic-dependent diseases as diabetic retinopathy^16^, peripheral vascular disease^17^, and cancers^18^.

Developing tailored treatment to angiogenesis-related diseases, such as in tumors or ischemia, can be enhanced by the use computational modeling. Computational modeling offers a systemic approach for optimizing ligand, receptor, and signaling targeting. It has predicted how anti-VEGF drug efficacy may depend on VEGFR1 concentrations on tumor endothelial cells^19^. It has provided predictions on anti-VEGF pharmacodynamics and pharmacokinetics in the body^13^. It has provided systematic analysis comparing the roles of heparin-binding and non-heparin-binding VEGF-A isoforms in tumors^20^. The advantage of these computational approaches is that predictions are defined by protein concentrations and kinetics (e.g. protein–protein-interactions). We can similarly apply computational modeling towards investigating how best to target these VEGF-A_xxx_b isoforms. Such models will require both quantitative insight into the relative concentrations for each isoform and also insights into their binding characteristics with the VEGF receptors, i.e. ligand:receptor kinetic rate constants and binding affinities.

Developing computational models requires precise measurements of the protein–protein interactions involved in the systems. Computational modeling has, therefore, been limited by VEGF-A splice variant kinetic binding data availability. More broadly speaking, precise measurements of ligand:receptor binding affinities and kinetics are crucial to understanding the systems at play: determining whether pro- or anti-angiogenic splice variants dominate the signaling axis is based both on the *ligand concentrations* and their *binding affinities*. Several models have incorporated the commonly-expressed VEGF-A_121a_ to study its role in physiological conditions, and several pathologies including breast cancers and peripheral artery disease (PAD)^21–26^. Current computational models have additionally evaluated anti-angiogenic VEGF-A_165b_ in diseases like PAD^27^. Each model operated under the assumption that VEGF-A_121a_ and VEGF-A_165b_ splice variants bind VEGF receptors with the same affinity as VEGF-A_165a_.

The conclusions reached, therefore, are highly dependent on whether VEGF-A splice variants truly bind their receptors with identical binding affinities. Therefore, measuring their precise kinetics to calculate their affinities is crucial to ensuring their predictions are accurate. Previously, only a small subset of the pro-angiogenic isoforms, VEGF-A_165a_, -A_121_, and -A_183_ have had their binding affinities measured^12,14^, and only VEGF-A_165a_ has *binding kinetic constants* (k_a_ and k_d_) measured^12^. The binding affinities (e.g., K_D_ = k_d_/k_a_) of the anti-angiogenic VEGF-A_165b_ and VEGF-A_165a_ to VEGFRs were previously compared *qualitatively* via a radiolabeled-ligand binding assay^14^, concluding that VEGF-A_165b_ and VEGF-A_165a_ bind with similar affinities, but also without providing quantitative measurements of the binding affinity (K_D_). However, these previous studies provided insufficient evidence for identical VEGFR binding affinities for two reasons: (1) Radiolabeled-ligand binding assays are unable to compare differences in ligand-binding kinetics constants (k_a_ and k_d_) and only provide binding data at equilibrium conditions (e.g., K_D_). However, no mathematical fittings were employed to quantify binding affinity K_D_, rendering it difficult to interpret the qualitative binding curves, which show VEGF-A_165a_ saturated VEGFR1 at a higher concentration than VEGF-A_165b_. Their results, suggest that VEGF-A_165b_ binds VEGFR1 with a stronger affinity, contrary to their reported conclusions. (2) The concentration range was limited. The lowest dose explored was sufficient to saturate VEGFR2 for both splice variants, preventing conclusive comparisons of their relative binding affinities. If the receptors start saturated, the results cannot conclude whether one ligand binds with a stronger affinity than the other, only that both ligands have affinities sufficient to saturate at those low-dose conditions. Differentiating whether VEGF-A_165b_ binds with identical affinity versus greater affinity than VEGF-A_165a_ would require testing doses *below* the receptor-saturating concentrations for both VEGF-A_165a_ and VEGF-A_165b_.

Surface plasmon resonance (SPR) is an ideal approach for addressing the unanswered questions of (a) whether VEGF-A_165a,_ VEGF-A_165b_, and VEGF-A_121_ bind VEGFR1 and VEGFR2 with identical binding affinities and (b) the kinetic rate constants for each interaction. SPR measures binding kinetics (k_a_ and k_d_) by detecting biomolecular interactions in real-time with high sensitivity^28,29^. Recent work has further advanced reference signal choice to minimize the detection of non-specific binding while measuring ligand:receptor kinetic constants for VEGF family receptors, specifically^30^. Recently, the SPR-based approach has been applied to compare pro- and anti-angiogenic binding properties, but these studies only qualitatively reported that the isoforms bound VEGFRs with binding affinities within two orders of magnitude of each other^12^, and these studies were performed before new reference subtraction methods were demonstrated^30^ . Kinetic rate constants have been reported only for VEGF-A_165_:VEGFR interactions^12,30,31^. These currently-unknown binding characteristics are needed to develop physiologically-accurate and predictive computational models.

Here we apply surface plasmon resonance (SPR) to measure the unknown VEGF-A_xxxa_:VEGFR1 and -A_xxxb_ binding kinetics. We observe differential binding that should lead to differential activation of the VEGFRs. These results will enable the construction of detailed, accurate computational models to help further our understanding of angiogenic signaling in human health and pathology.

## Results

### Establishing VEGFR binding kinetics for VEGF-A splice variants

**Figure 2:**
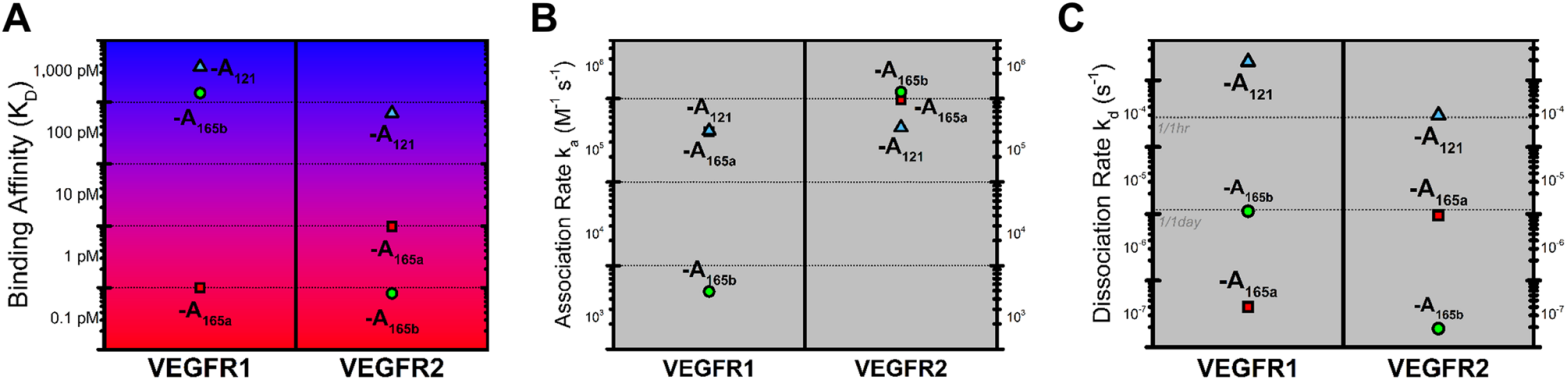
Binding kinetics and affinities for VEGFR1 and VEGFR2 interactions with VEGFA splice variants. (**A**) Binding affinity K_D_ between each ligand and receptor, (**B**) association rate (in M^−1^ s^−1^) and (**C**) dissociation rate (s^−1^).

**Figure 3:**
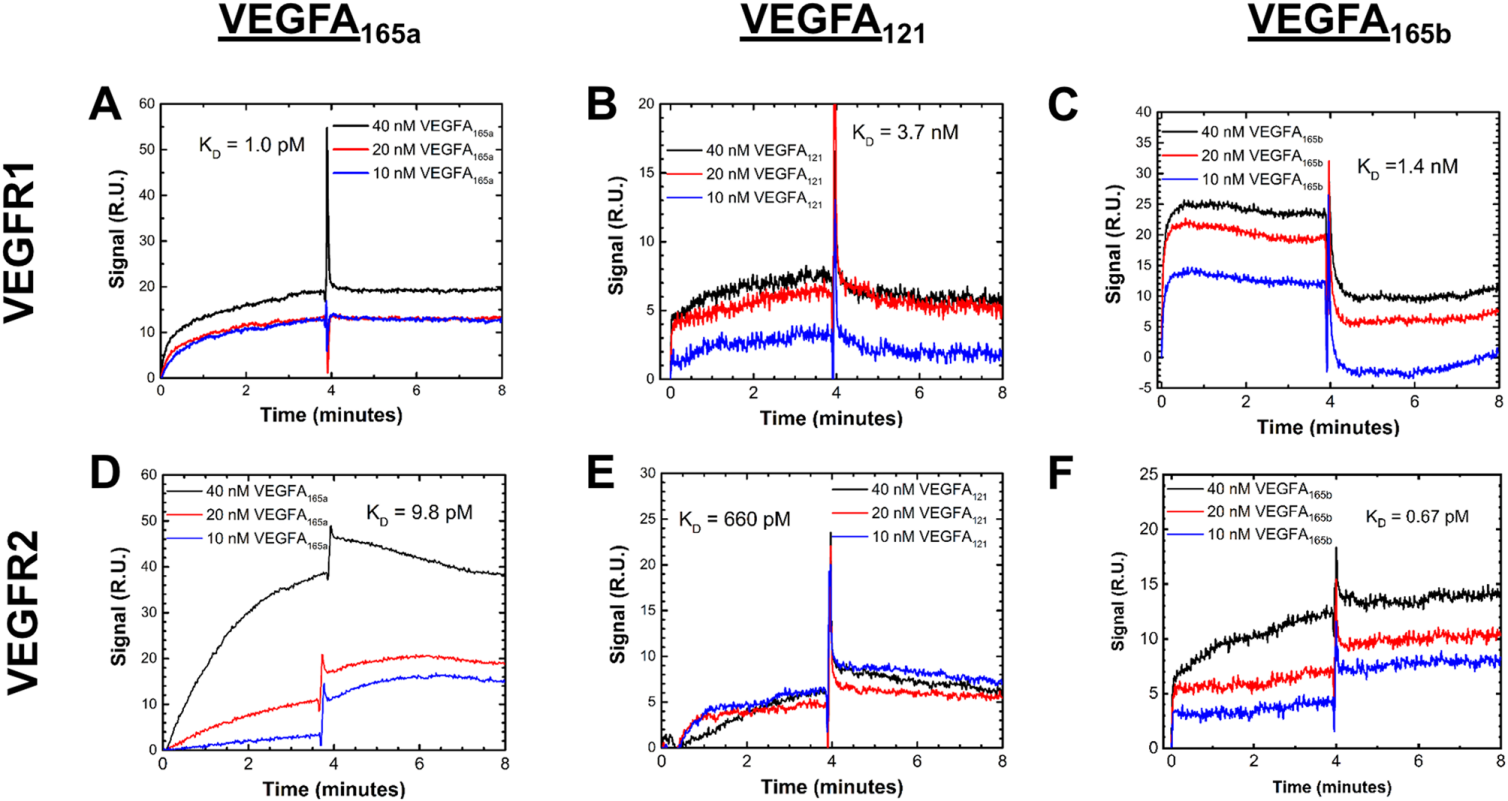
SPR sensograms for VEGFA_165a_, -A_121_, and -A_165b_ binding VEGFR1 and VEGFR2. (**A**) Kinetic response curve of VEGFA_165a_–VEGFR1 and (**D**) VEGFR2 binding confirmed previous high affinity measurements to both receptors. (**B**) Both VEGFA_121_ and the anti-angiogenic VEGFA_165b_ variants bind VEGFR1 with affinities 3 orders of magnitude weaker than VEGFA_165a_. (**E**) VEGFA_121_ binds VEGFR2 with the weakest affinity, and (**F**) the anti-angiogenic VEGFA_165b_ binds VEGFR2 with a strong affinity that could displace the angiogenic form. Receptors were immobilized on BIAcore CM5 sensors. All kinetic studies were obtained by simultaneously injecting ligand across VEGFR1, VEGFR2, and Angiopoietin-4, a non-VEGFA binding protein used for reference subtraction. The following curves were obtained by subtracting this reference signal from the raw receptor-ligand interaction curve, removing signals associated with non-specific interactions.

We utilized the SPR biosensor-based assay to measure the unknown kinetics for VEGF-A_121_ and VEGF-A_165b_ binding to VEGFR1 and VEGFR2. We confirmed that the canonical view of VEGFR:VEGF-A splice variant binding holds: VEGF-A_165a_, -A_165b_, and -A_121_ bind both VEGFR1 and VEGFR2^32^. We validated the method with VEGF-A_165a_ binding kinetics^30,31^. We measured a VEGF-A_165a_:VEGFR1 binding affinity (Fig. 2A; and Supplementary Table S1) binding affinity of K_D_ = 1 pM (Fig. 3A); which is consistent with a prior SPR affinity of K_D_ = 7.5 pM^31^. We measured a VEGF-A_165a_:VEGFR2 binding affinity of K_D_ = 9.8 pM, which is within an order of magnitude of a previous SPR measurement found by Tiedemann et al. (K_D_ = 52 pM^33^). We observe that VEGFA_165a_ binds VEGFR1 with 3-orders of magnitude stronger affinity than either the anti-angiogenic -A_165b_ or -A_121_: VEGF-A_165a_ binds VEGFR1 with K_D_ = 1.0 pM. VEGF-A_165b_, however, binds VEGFR1 with a K_D_ = 1.4 nM. VEGF-A_121_ binds VEGFR1 with the weakest affinity of the three isoforms with a K_D_ = 3.7 nM. (Fig. 2A, and Supplementary Table S1). In contrast, we observe that the -A_165b_ variant binds VEGFR2 10 × stronger than the -A_165a_ variant with a VEGF-A_165b_:VEGFR2-affinity constant of K_D_ = 0.67 pM versus a VEGF-A_165a_:VEGFR2 K_D_ = 9.8 pM (Fig. 2A, Supplementary Table S1). In contrast, we observe VEGFA_121_:VEGFR2 binding occurred with the weakest strength, with an affinity constant K_D_ = 660 nM (Fig. 2A). These binding affinities disagree with recent SPR studies of VEGF-A_121_:VEGFR1 and VEGFR2^34^. In their studies, however, Teran et al. also report nM affinities for the well-established picomolar affinities measured for VEGF-A_165a_ to VEGFR1 and VEGFR2. We attribute these discrepancies to their use of FGFR1 as a receptor reference protein. Structurally similar receptor proteins enable greater non-specific binding with structurally similar ligands^35^, thereby allowing an overestimated reference signal, underestimating the binding affinities. Alternatively, VEGF-A_165a_:FGFR1 may specifically bind cross-family, similar to the PDGF:VEGFR2 binding which was recently demonstrated to occur^30^.

### Association and dissociation rate constants reveal affinity trends

We can further understand the VEGF-A_xxx_:VEGFR interaction affinities via their association and dissociation rate constants (Fig. 2B, C) obtained from analyzing the SPR sensograms (Fig. 3A–F). Here we observe that the difference in -A_165a_ and -A_165b_ binding affinities with VEGFR2 is attributed to their dissociation kinetics. VEGFA_165a_ and VEGF-A_165b_ bind VEGFR2 with similar association rates—i.e. k_a_ ≃ 10^6^ M^−1^ s^−1^; however, A_165a_:VEGFR2 has a dissociation rate 50-fold faster than does -A_165b_:VEGFR2, resulting in the higher binding affinity between the latter pair (Fig. 3B, C). In contrast, -A_165b_:VEGFR1 dissociates ~ 30-fold faster than does VEGF-A_165a_:VEGFR1, but with a, -A_165b_:VEGFR1 association rate ~ 2 orders of magnitude slower than for VEGF-A_165a_:VEGFR1 k_a _≃ 4.9 * 10^3^ versus 4.0 * 10^5^). Together this results in a 1,000-fold weaker -A_165b_:VEGFR1 interaction (Fig. 3A–C).

In contrast to the variation in dissociation rates, we observed minor association rate variation across VEGF-A_121_, -A_165a_, and -A_165b_. For both VEGFR1 and VEGFR2, the splice variants association rates varied within an order of magnitude. VEGF-A_121_ bound VEGFR1 with an association rate within an order of magnitude of VEGF-A_165a_ and -A_165b_. Likewise, all three variants bound VEGFR2 with association rates within an order of magnitude (Fig. 3B).

## Discussion

We have shown two key findings: (1) differential binding between VEGFRs and the three most common^36–38^ VEGF-A splice variants, VEGF-A_165a_, VEGF-A_121_, and VEGF-A_165b_; and (2) we measured the kinetics and affinities for each interaction. The differential VEGFR binding may be key to understanding VEGF-A_xxx_ protein expression patterns in numerous pathologies, including peripheral artery disease (PAD), cancers, obesity, and other disorders like systemic sclerosis^39–43^.

### Differential VEGF-A_xxx_:VEGFR binding kinetics in VEGFR signaling

Our measurements for VEGF-A_xxx_:VEGFR binding fill an important gap in our understanding of splice variant interactions with their receptors. Previous work had measured binding affinities for a subset of the “pro-angiogenic” VEGF-A_xxxa_ isoforms—VEGF-A_165a_, -A_121_, and -A_183_, but precise binding kinetics were found previously only for -A_165a_^31^. Additionally, the binding affinities were only qualitatively compared between the weak-agonist VEGF-A_165b_ and VEGF-A_165a_ to VEGFRs via a radiolabeled-ligand binding assay^14^. These studies concluded that VEGF-A_165b_ and VEGF-A_165a_ bind with similar affinities, without providing quantitative measurements of the binding affinity (K_D_) nor providing binding kinetics necessary to model their dynamics computationally. Our results address this gap by measuring the binding kinetic constants, under experimental conditions that reflect the relative ligand and receptor expression levels found physiological-comparable conditions, for VEGF-A_121_ and VEGF-A_165b_, revealing stronger VEGF-A_165b_:VEGFR2 binding versus -A_165a_, and overall weaker VEGF-A_121_:VEGFR binding.

The kinetic measurements in these studies focused on the three most prevalent VEGF-A splice variants^36–38^, however, several other human splice variants still require precise kinetic and affinity quantification measurements, such as VEGF-A_189_, and the other -A_xxxb_ variants besides VEGF-A_165b_. The systemic experimental approach here provides a framework for approaching this task. For example, this can be used to understand splice variant binding to the co-receptor neuropilin-1 and heparin sulfate (HS) chains surrounding the cell, since they are known to binddifferentially^11,44,45^. The VEGF-A_xxx_b variants specifically have a diminished capacity to bind neuropilin-1 and HS-chains, preventing their cooperative binding to VEGFR2^14^. These phenomena may explain differences between the results reported here and studies^46^ by Ganta et al. This prior work reported VEGF-A_165b_ outcompeting VEGF-A_165a_, for VEGFR1 binding, via qualitative competitive binding studies on endothelial cells. In contrast, we directly measure binding to isolated recombinant receptors. The difference between the *recombinant* VEGF-A:VEGFR binding affinities versus the in vitro* binding* to cell-bound receptors likely reflects these differential interactions between VEGF-A_165x_ splice variants with co-receptors and ECM components, as these alternate binding partners will sequester splice variants to different degrees^11,44,45^. Measuring the ‘effective’ binding affinities in their presence could be accomplished by performing SPR experiments in the presence of neuropilins, heparins, and glycoproteins such as fibronectin, or by performing SPR directly on cells using a number of approaches recently reviewed^34^. The differences between the strength of these interactions, coupled with the differential ligand:receptor binding affinities, will need to be considered together to predict precisely the physiological role for these splice variants.

Towards further understanding the significance of additional binding partners, it will be key to quantitatively measure NRP1 and HS-chain abundance. Quantification of NRP1 would indicate that it could significantly sequester VEGF-ligands, with NRP1 ranges of 40,000–68,000 on the plasma membrane of a human blood or lymphatic endothelial cell, in vitro^47,48^ and over 100,000 membrane NRP1 per fibroblast, in vitro^47^. Moreover, HS chain estimates of ~ 1 per NRP1 on endothelial cells, and ~ 1–4 on basement membrane proteoglycans^49^, further demonstrate the significance of HS significance. Such protein measurements can be obtained using quantitative flow cytometry^50^, and could further compare how NRP1 and ECM expression differ across physiological and pathological states. In silico work has predicted that NRP1 can significantly sequester VEGFs in the pathological condition of cancer and in doing so, improve anti-VEGF efficacy (decreased free-VEGF). Two conditions were predicted to enable this improved therapeutic efficacy: (1) increased numbers of NRP1 on cells^51^ or (2) inhibition of VEGFR2–NRP1 interactions^52^. Additional explorations of NRP1 or HS-chain effects can be achieved via modeling, and a recent model provided an accessible guide for understanding for how concentrations and binding kinetics would alter the signaling landscapes^53^. However, future studies would require measurement of VEGF-NRP1 and VEGF-HS kinetics to faithfully evaluate how they alter angiogenic responses.

Additionally, our measurements of differential binding between VEGF-A splice variants stress the importance of understanding how each splice variant effects different responses *downstream* of ligand:receptor binding. While ligand-receptor binding is a key first-step in RTK activation, the resulting receptor conformational changes induced are critical in allowing tyrosine residue phosphorylation^5^. Therefore, different VEGF-A splice variants can induce receptor dimer confirmations that selectively inhibit the activity of some tyrosine residues while promoting phosphorylation at other residues. This is best exemplified by VEGF-A_165b_, which can promote comparable VEGFR2^Y1175^ phosphorylation while inhibiting VEGFR2^Y1052^ phosphorylation^54^. In contrast, VEGF-A_165b_:VEGFR1 binding has been shown to inhibit VEGFR1^Y1333^ phosphorylation^46^. Since, VEGFR functional responses are regulated by differential tyrosine residue phosphorylation^55–57^, it is critical that future work fully characterize the signaling elicited by each splice variant.

Our results, demonstrating that VEGF-A_165b_ binds VEGFR2 selectively with stronger affinity than -A_165a_, may help explain why isoform specific up-regulation and down-regulation have either pro- or anti-angiogenic results in different pathologies. This is based on existing views that VEGF-A_165b_ does not induce significant signaling through its binding to VEGFR2. For instance, Kawamura et al.^54^ demonstrated VEGF-A_165a_ treatment, at receptor-saturation levels, resulted in *reduced* VEGFR2 phosphorylation levels. However, it is critical to note that recent evidence suggests that the both so-called “pro-” and “anti-angiogenic isoforms”, VEGF-A_165b_ and -A_165a_, can phosphorylate VEGFR2^Y1175^ in peripheral artery disease^46^. Ganta et al.^46^ demonstrated that inhibiting VEGF-A_165b_ did not result in significantly increased activity at VEGFR2^Y1175^, suggesting that -A_165b_ was not acting as a full antagonist at this VEGFR2 residue under non-receptor saturating conditions. Taken together, the action of VEGF-A_165b_ depends on the concentration of VEGF-A_165a_: high-affinity binding of VEGF-A_165b_ to VEGFR2 would result in a significant decrease in VEGFR2 activity when VEGF-A_165a_ concentrations saturate VEGFR2.

While research is currently limited for VEGF-A_165b_-induced VEGFR2 phosphorylation, it is possible that VEGF-A_165b_ may play a partial agonist role at *some* tyrosine residues, while inhibiting other pro-angiogenic tyrosine sites. .Indeed, a recent review concludes that VEGF-A_165b_ ultimately acts as a less efficacious agonist, and only at some residues^15^. From this evidence, one can speculate that VEGF-A_165b_ acts with *functional selectivity,* more commonly known as biased agonism. This is a common phenomenon in G-coupled protein receptor signaling whereby different ligands, binding the same receptor, promote activation of different downstream signaling pathways^58–60^. Therefore, under biased agonism, VEGF-A_165b_ may act as an agonist at certain tyrosine residues while acting antagonistically at others.

### Conclusion

Alternative splicing produces VEGF-A variants with different binding partners, and as we show for the first time, different binding kinetics. As recent evidence has pointed to important roles the pro- and anti-angiogenic variants can play in human pathology, it is crucial for researchers to not only have access to differences in their expression, but these differences we have identified in their binding kinetics. Cell signaling systems, like the VEGF-A-axis regulating angiogenesis, are complex: receptors activate a multitude independent and overlapping downstream pathways, with dozens of growth factors binding receptors, either activating, inhibiting, or modulating their downstream action. Deconvolving the contributions of any one molecule requires the experimental tools provided by systems biology. To this end, further work remains, in characterizing the binding, receptor activation, and downstream biological effect produced by other ‘anti-angiogenic’ VEGF-A_xxxb_ splice variants, such as VEGF-A_121b_—which have hitherto received less investigation^12,15,61^, as well as measuring differences in splice variant binding to the ECM, heparin sulfates chains, and their associated proteoglycans, and co-receptors like neuropilins^17,46^. By having a more complete picture of the VEGF-A splice variants will we be able to advance more effective therapeutic interventions for angiogenesis-related pathologies.

## Materials and methods

### Surface plasmon resonance kinetic studies with dextran-coated gold sensors

SPR studies were performed with the BIAcore 3,000 instrument (Biacore International AB, Uppsala, Sweden) at 25 °C on dextran-coated gold sensor chips (CM5, Research grade, GE Healthcare Bio-sciences AB, Uppsala) following a previously established procedure^30^. The BIAcore 3,000 divides CM5 sensor chips into four separate flow cells. We immobilized a different receptor protein in each flow cell: The first cell was reserved for measuring non-specific binding by immobilizing recombinant angiopoietin-4 (Cat. #964-AN-025/CF, R&D Systems) to a flow cell: it has no known interaction with VEGF-A splice variants. VEGFR1 and VEGFR2 were immobilized in two of the remaining flow cells. *Running buffer*: 1 × HBS-EP pH 7.4 (10 mM HEPES, 3 mM EDTA, 150 mM NaCl, 0.005% TWEEN-20, cat. # BR100188, GE Life Sciences).

### Protein immobilization

Pre-concentration studies were performed to determine optimal pH conditions for protein immobilization to ensure target levels of immobilized protein can be achieved precisely and to conserve protein. 10 mM acetate buffers were prepared ranging from pH 3.5 to 0.5–1.0 below the protein’s isoelectric point (Supplementary Table S1). Receptor solutions were prepared at 20 µg/mL with each acetate buffer. We injected 20 µL of each pH solution at a flow rate of 5 µL/min, followed by a 5-µL injection of ethanolamine-HCL (GE Healthcare AB, Uppsala, Sweden) to clear the surface. We selected the optimal acetate buffer pH for each protein based on (1) the maximum level of protein immobilization reached and (2) the rate of immobilization observed in the pre-concentration study sensograms.

Recombinant VEGFR1 (Recombinant Human VEGF R1/Flt-1 Fc Chimera Protein, CF, Cat. #321-FL-050/CF, R&D Systems) and VEGFR2 (Recombinant Human VEGF R2/KDR Fc Chimera Protein, CF, Cat. #357-KD-050/CF, R&D Systems) were immobilized irreversibly to separate flow cells via amine coupling with the dextran matrix. Additionally, we immobilized angiopoietin-4 (Recombinant Human Angiopoietin-4 Protein, CF, Cat. #964-AN-025/CF, R&D Systems), a protein with no known interaction with any VEGF-A splice variant, to a flow cell as a reference signal protein to subtract the effects of non-specific interactions. The surface was activated by injecting 35 μL of a 1:1 volumetric mixture of 0.05 M NHS (N-hydroxysuccinimide, GE Healthcare AB) and 0.2 M EDC (1-ethyl-3-(3-dimethylaminopropyl) carbodiimide hydrochloride GE Healthcare AB) at a flow rate of 5 μL/min. Each receptor was dissolved in 20 µg/mL of the 10 mM acetate buffer at its optimal pH and injected at 5 µL/min until the target level was reached (approximately 200–500 R.U. of immobilized receptor, preventing quick receptor saturation at injected ligand concentrations.) After sufficient protein was coupled, the surface was de-activated by injecting 35 μL ethanolamine (ethanolamine hydrochloride-NaOH pH 8.5, GE Healthcare AB).

### Ligand-receptor kinetics measurements

Fresh ligand solutions were prepared across a range of ligand concentrations in HBS-EP running buffer—10 nM, 20 nM, and 40 nM—each experimental day, including: human recombinant VEGFA (R&D Systems, Cat. #293-VE-010), VEGFA_121_, and VEGFA_165b_. These concentration ranges were selected such that the injected concentrations exceeded the immobilized receptor concentrations, which enables these experiments to best reflect the physiological reality, and follow the configuration of recent computational models^27^. 120 μL of each ligand solution was injected into flow cells containing immobilized receptor and Ang-4—a reference for non-specific binding—at 30 μL/min (association). This was followed by a 10 min running-buffer injection (dissociation). Between each sample, we injected, in series, 5 μL of 5 mM HCl and 5 μL of 10 mM NaOH at 5 μL/min to remove any remaining bound ligand. We repeated this cycle for each concentration tested (40, 20, and 10 nM). Each concentration series were performed in triplicate. All recombinant human cytokines were obtained from R&D Systems.

### Kinetic analysis

The raw ligand:receptor sensograms were aligned and the background, non-specific binding was subtracted using the sensogram trace from the ligand:Ang-4 flow cell. Both the raw ligand:receptor and ligand:Ang-4 sensogram curves were obtained within the BIAcore 3,000’s detection window (10–70,000 R.U.) for all interactions^62^ to ensure detected interactions did not represent system noise. BIA evaluation removes momentary signal spikes resulting from transient air bubbles.

Global analysis is considered to produce more accurate results than fitting of a single response curve, so global fitting was performed with BIAevaluation software (Version 4.1.1, GE Healthcare) following a 1:1 Langmuir adsorption isotherm (Eq. 1)^63^. The software applies nonlinear least squares analysis to determine association (*k*_*a*_) and dissociation (*k*_*d*_) rates fitting best to multiple response curves simultaneously. Additionally, the software provides the goodness-of-fit parameter χ^2^ and the peak magnitude of the signal response, R_max_.

1 $$ R + L\mathop \leftrightarrow \limits^{{k_{a} ,k_{d} }} RL $$

### Classifying binding

Both the instrument manufacturer (BIAcore) and previous researchers have suggested that when fitting kinetic rate constants using global analysis, a χ^2^-to-R_max_ value (a measure of noise-to-signal) < 0.2 is ideal for confidence in the kinetic parameters obtained when studying known interactions^64–66^. A low noise-to-signal indicates that the sensogram signal includes minimal contributions from the following three noise-factors: (1) overall instrument noise, (2) heterogeneities in immobilized receptor or ligand, and (3) non-specific interactions. We applied a cut-off value we established previously^30^ of χ^2^-to-R_max_ < 1.0 that differentiates real, 1:1 Langmuir interactions, from predominantly non-specific interactions, where χ^2^-to-R_max_ > 1.0 to confirm that detected interactions represented true receptor binding. (χ^2^-to-R_max_ values obtained are summarized in Supplementary Table S2).

## Supplementary information

Supplementary Tables.
