# Supplementary material for: VEGF-A splice variants bind VEGFRs with differential affinities
Source: Sci Rep. 2020 Sep 2;10:14413. doi: 10.1038/s41598-020-71484-y (PMC7468149; doi:10.1038/s41598-020-71484-y)
Supplement: Supplementary file 1 — Supplementary Tables. [file 41598_2020_71484_MOESM1_ESM.pdf]

VEGF-A splice variants bind VEGFRs with differential affinities

Authors: Spencer B. Mamer[1*], Ashley Wittenkeller[1], and P. I. Imoukhuede[2]

[1]Department of Bioengineering, University of Illinois at Urbana-Champaign, Urbana, IL
[2]Department of Biomedical Engineering, Washington University in St. Louis, St. Louis, MO
*Correspondence to: smamer2@illinois.edu

Supplementary Tables

Supplementary Table S1. VEGF-A_{xxx}:VEGFR binding kinetics and affinities. Kinetic constants were obtained by performing global kinetic analysis using the BIAevaluation software across binding sensograms obtained at 10 nM, 20 nM, and 40 nM for each ligand-receptor pair. Affinity constants were calculated as the ratio of the association and dissociation rates. Rate constants where the χ^2-to-R_{max} ratio exceeds 1.0 are shown *in italics*, indicating interactions that are dominated by non-specific interactions. Ligand-receptor pairs that produced negative association curves were not analyzed with global fitting (indicated by "NI" for no interaction). All values are presented as the arithmetic mean ± standard error of the mean (SEM). (*$p < 0.05$, **$p < 0.01$, ***$p < 0.001$; each concentration series was performed in triplicate).

	**VEGFR1**			**VEGFR2**		
	$\mathbf{k_a}$ (M^{-1} s^{-1})	$\mathbf{k_d}$ (s^{-1})	$\mathbf{K_D}$ (M)	$\mathbf{k_a}$ (M^{-1} s^{-1})	$\mathbf{k_d}$ (s^{-1})	$\mathbf{K_D}$ (M)
-A_{165}	$4.0 \pm 0.04 * 10^{5}$	$4.0 \pm 0.1 * 10^{-7}$	$1 \pm 0.3 * 10^{-12}$	$9.7 \pm 0.3 * 10^{5}$	$9.5 \pm 0.2 * 10^{-6}$	$9.8 \pm 0.4 * 10^{-12}$
-A_{121}	$4.1 \pm 0.3 * 10^{5}$	$1.9 \pm 0.07 * 10^{-3}$	$3.7 \pm 0.3 * 10^{-9}$	$4.5 \pm 1.4 * 10^{5}$	$3.0 \pm 0.5 * 10^{-4}$	$6.6 \pm 2.0 * 10^{-10}$
-A_{165b}	$4.9 \pm 0.8 * 10^{3}$	$1.1 \pm 0.3 * 10^{-5}$	$2.3 \pm 0.7 * 10^{-9}$	$1.2 \pm 0.9 * 10^{6}$	$9.7 \pm 0.1 * 10^{-7}$	$8.1 \pm 0.6 * 10^{-13}$

Supplementary Table S2. χ^2-to-R_{max} quality-of-fit for $VEGFA_{xxx}$:VEGFR global kinetic fitting. The ratio reflects how well a global kinetic analysis of a multi-concentration sensogram series can fit to a simple 1:1 Langmuir interaction model can fit. True interactions were distinguished from those due to non-specific binding patterns where χ^2-to-R_{max} < 1.0 (indicated by green shading and polka dot texturing). Fitted curves where χ^2-to-R_{max} > 1.0 (in red-shaded, grid-pattern textured cells) were considered to be dominated by non-specific binding. Ligand-receptor pairs that produced negative association curves were not analyzed with global fitting (indicated by "NI" for no interaction).

	**VEGFR1**	**VEGFR2**
$-A_{165}$	0.17	0.52
$-A_{121}$	0.13	0.029
$-A_{165b}$	0.039	0.12
